# WAXD, polarized ATR-FTIR and DSC data of stress-annealed poly(3-hydroxybutyrate) fibers

**DOI:** 10.1016/j.dib.2021.107523

**Published:** 2021-10-27

**Authors:** E. Perret, K. Sharma, S. Tritsch, R. Hufenus

**Affiliations:** a Laboratory for Advanced Fibers, Empa, Swiss Federal Laboratories for Materials Science and Technology, Lerchenfeldstrasse 5, 9014 St. Gallen, Switzerland; b Center for X-ray Analytics, Empa, Swiss Federal Laboratories for Materials Science and Technology, Überlandstrasse 129, 8600 Dübendorf, Switzerland; c KTH Royal Institute of Technology, Stockholm, 114 16 Sweden; d Hochschule Reutlingen, Alteburgstrasse 150, 72762 Reutlingen, Germany

**Keywords:** Wide-angle x-ray diffraction, Polarized attenuated total reflection Fourier Transform infrared spectroscopy, P3HB, poly(3-hydroxybutyrate), Biodegradable, Melt-spun, Stress annealing, Fiber

## Abstract

This article summarizes synchrotron wide-angle x-ray diffraction (WAXD) patterns, polarized attenuated Fourier transform infrared spectroscopy (ATR-FTIR) data and differential scanning calorimetry (DSC) data of differently stress-annealed poly(3-hydroxybutyrate) (P3HB) fibers. Additionally, in-situ polarized ATR-FTIR data has been measured under tensile drawing of pre-annealed P3HB fibers under low annealing stress. Modifications to the ATR-FTIR setup and sample holders for performing measurements on P3HB fibers are explained in the experimental section. For more information see ‘Reversible mesophase in stress-annealed poly(3-hydroxybutyrate) fibers: A synchrotron x-ray and polarized ATR-FTIR study' [1].

## Specifications Table

**Table:** 

Subject	Materials Science: Polymers and Plastics
Specific subject area	Biodegradable melt-spun monofilaments.
Type of data	Table Equation Image Figure
How data were acquired	Instruments: Synchrotron X-ray measurements (cSAXS beamline, PSI, Switzerland) Bruker Tensor 27 FTIR spectrometer(Bruker Optics, Ettlingen, Germany) Attenuated total reflectance (GladiATR™) accessory from Pike Technologies (Fitchburg, Wisconsin, United States) DSC 214 Polyma (Netzsch, Selb, Germany). Software: Matlab R2018a Python codes
Data format	Raw Analyzed
Parameters for data collection	Synchrotron wide-angle x-ray diffraction (WAXD) patterns were taken of differently stress-annealed melt-spun poly(3-hydroxybutyrate) P3HB fibers. Polarized attenuated total reflection Fourier transform infrared (ATR-FTIR) spectroscopy data was collected of differently stress-annealed melt-spun P3HB fibers. Polarized ATR-FTIR was also performed in-situ under tensile drawing of pre-annealed fibers. Differential scanning calorimetry was performed on P3HB fiber pieces.
Description of data collection	Synchrotron WAXD measurements were performed at the cSAXS beamline at the Swiss Light Source of the Paul Scherrer Institute in Switzerland. P3HB fibers have been mounted on a sample holder and WAXD patterns were measured with 5 second exposures using a Pilatus 2M detector. The sample to detector distance was 31.9 cm. The x-ray beam was focused with mirrors to a spot size of about 10 µm (perpendicular to fiber axis) and its energy was set to 11.792 keV. Polarized ATR-FTIR spectra have been recorded from P3HB fibers with a Bruker Tensor 27 FTIR spectrometer (Bruker Optics, Ettlingen, Germany), using a single reflection attenuated total reflectance (GladiATR™) accessory from Pike Technologies (Fitchburg, Wisconsin, United States). Setup modifications are described in this article. Differential scanning calorimetry data was collected of differently stress-annealed P3HB fibers, by cutting the fibers into pieces and measuring with the instrument DSC 214 Polyma (Netzsch, Selb, Germany).
Data source location	Empa, St. Gallen, Switzerland
Data accessibility	Mendeley Data DOI: 10.17632/cpdyrdfx9y.1
Related research article	*E. Perret, K. Sharma, S. Tritsch, R. Hufenus, Reversible mesophase in stress-annealed poly(3-hydroxybutyrate) fibers: A synchrotron x-ray and polarized ATR-FTIR study, Polymer 231 (2021) 124141.* https://doi.org/10.1016/j.polymer.2021.124141

## Value of the Data

• Structural changes in biodegradable and biocompatible P3HB fibers due to stress-annealing and post tensile drawing strongly affect physical properties of the fibers and the presented data is therefore of value for biomedical applications.
• Mesophase content dependency on annealing conditions strongly affects mechanical properties of P3HB fibers and is therefore of interest to the textile and medical sector.
• The detailed description of ATR-FTIR setup modifications for polarized measurements of thin polymer fibers is of interest to other scientists, since the presented method can be applied to other types of fine filaments.
• Polarized ATR-FTIR data of stress-annealed and post-drawn P3HB fibers gives a better understanding of structural changes and is of interest to the textile community.
• The use of DSC to study the crystallinity of differently annealed P3HB fibers is of interest to other researchers, since the same method can be applied to study the crystallinity in other polymer fibers.

## 1 Data Description

### 1.1 Synchrotron WAXD analysis

#### 1.1.1 Absence of mesophase off-axis reflections in WAXD patterns of P3HB fibers

**Fiber (II)**

**Fig. 1:**
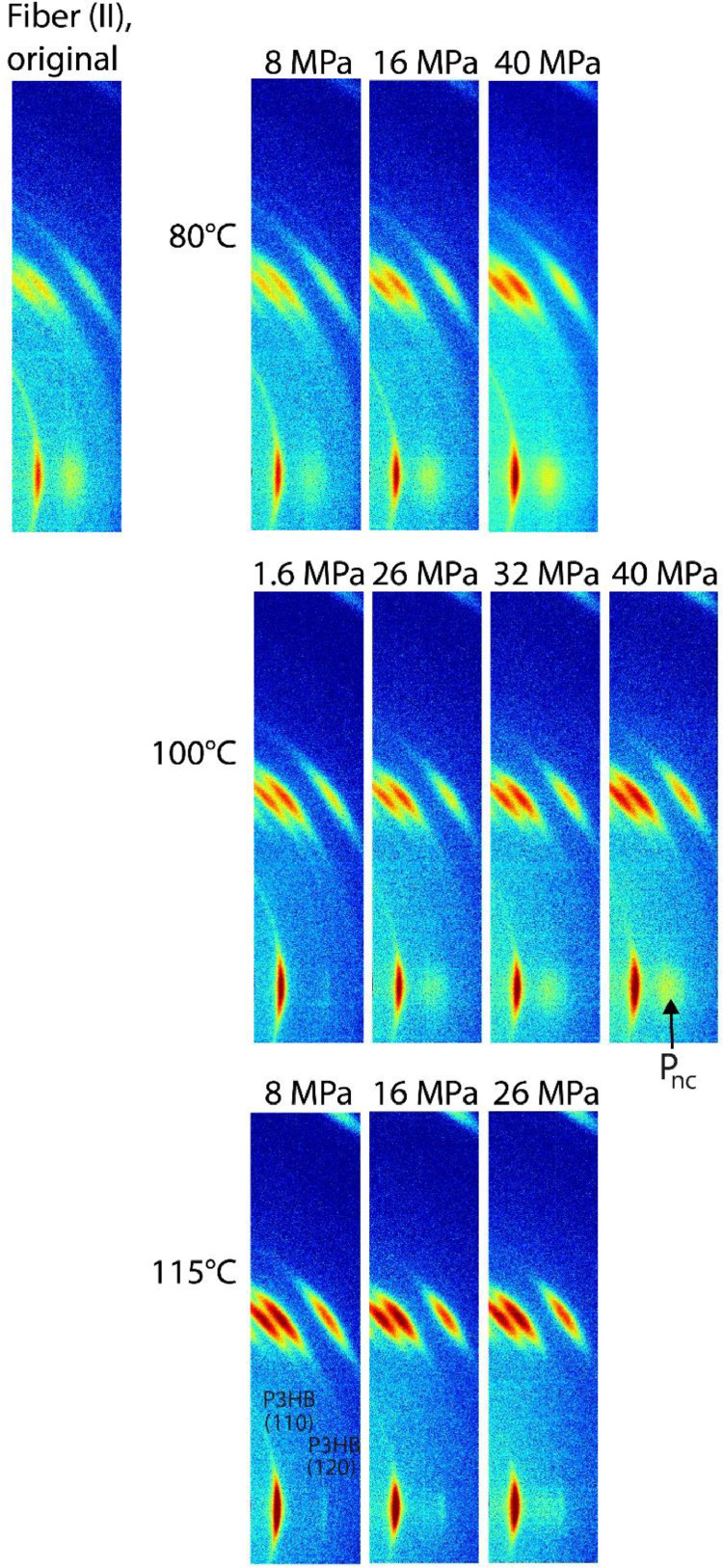
Close-ups of WAXD patterns measured at the synchrotron with 5s exposures of stress-annealed fiber (II) at 80°C (top row), at 100°C (middle row) and at 115°C (bottom row) for different applied stress values.

Fig. 1 shows close-ups of WAXD patterns around the equatorial mesophase peak, as well as around the first layer line, for the as-spun fiber (II) and stress-annealed fibers at different temperatures and for different applied stresses. No off-axis reflections are observed above the first layer line. The mesophase content is higher for high-stress annealed fibers, and for low-stress annealed fibers it also shows a higher mesophase content for lower temperatures. Note that all WAXD (.cbf) images from the Mendeley repository can all be plotted with the open source Python package from the Paul Scherrer Institute (https://github.com/paulscherrerinstitute/cbf).

#### 1.1.2 Mesophase content dependency on annealing conditions

**Fiber (I)**

**Fig. 2:**
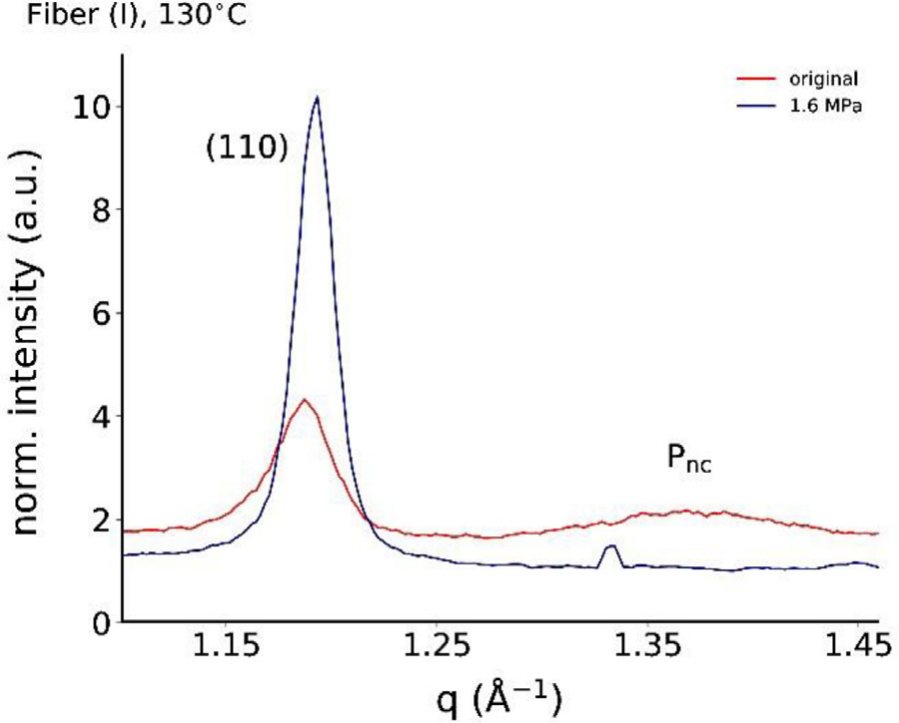
Equatorial profiles for stress-annealed fiber (I) at 130°C.

The equatorial profiles of as-spun fiber (I) and annealed fiber (I) at 130°C under low stress (1.6 MPa) are shown in Fig. 2.

**Fiber (II)**

**Fig. 3:**
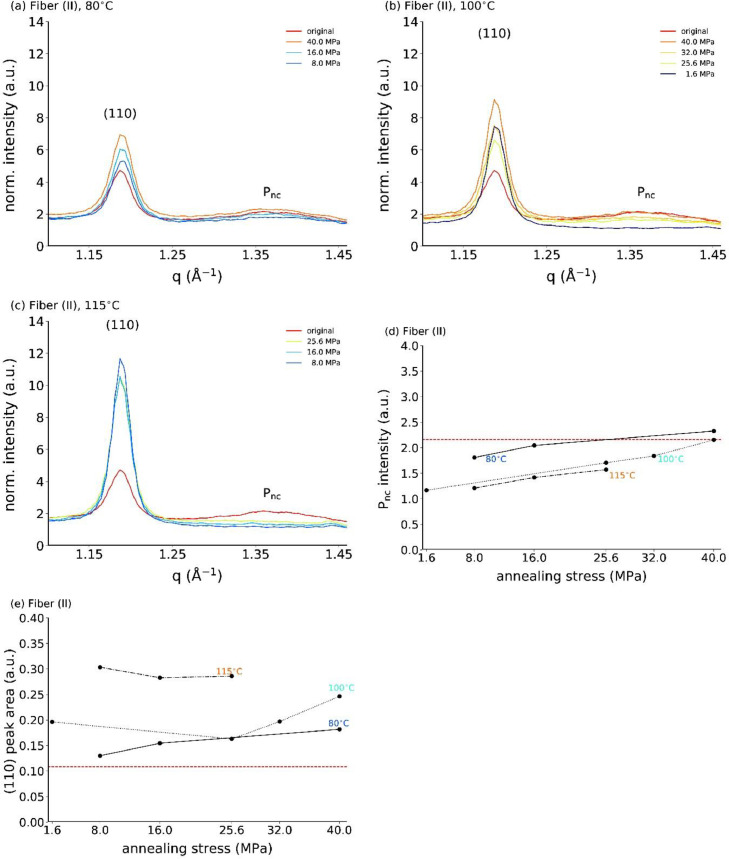
Equatorial profiles for stress-annealed fiber (II) at (a) 80°C, (b) 100°C and (c) 115°C. The corresponding mesophase peak intensities are shown in (d), and (110) peak areas are shown in (e), as a function of annealing stress. The dashed horizontal red lines in (d) and (e) are the reference values of the original fiber (II).

Fig. 3 summarizes all profiles for the stress-annealed fiber (II) at various temperatures (80, 100, 115°C). Fig. 3d shows the dependence of the mesophase peak intensity as a function of annealing stress, and Fig. 3e the one of the (110) peak area.

### 1.2 Polarized ATR-FTIR analysis

#### 1.2.1 Mesophase content dependency on annealing conditions

**Fiber (I)**

**Fig. 4:**
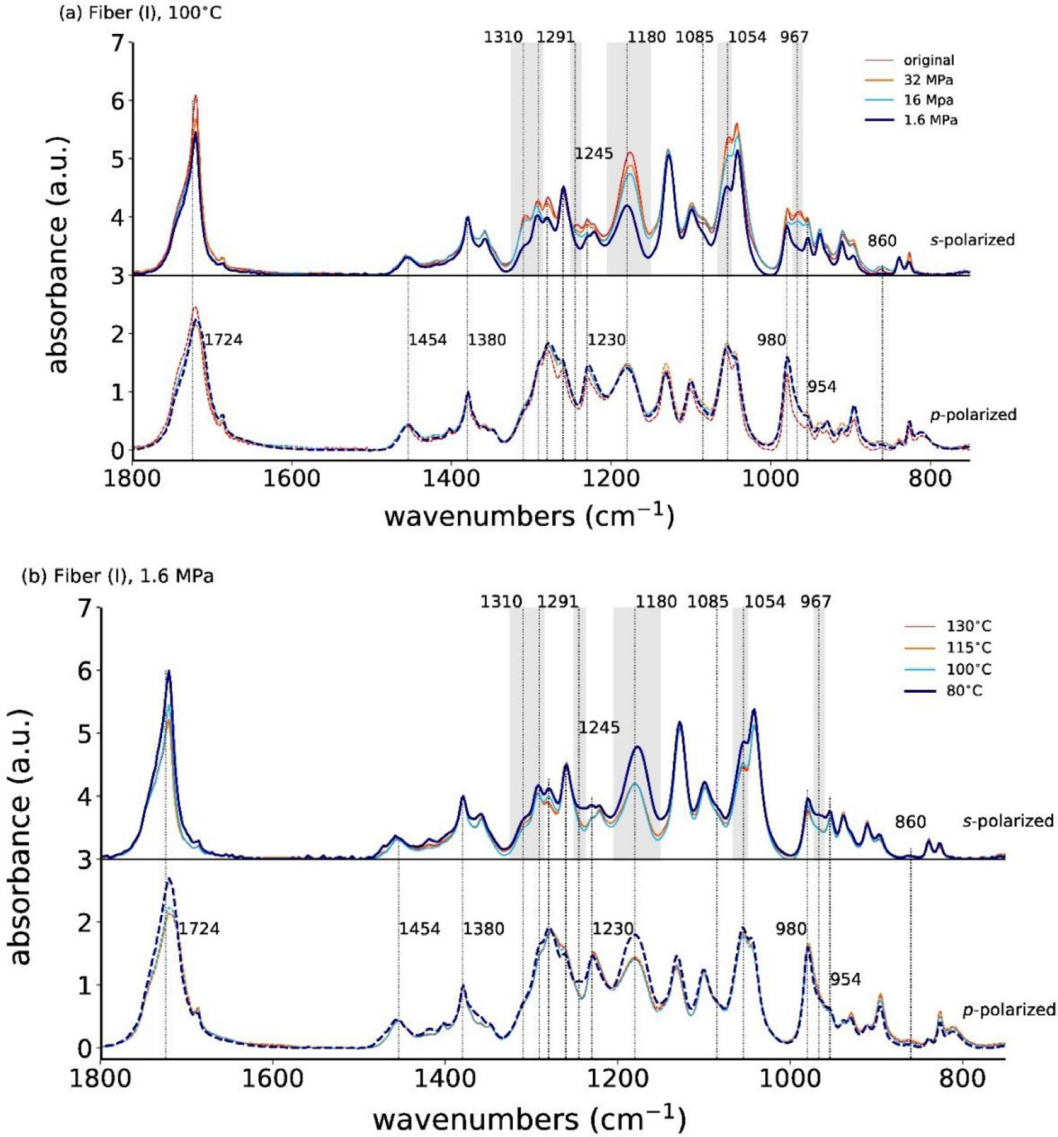
Polarized ATR-FTIR spectra for the as-spun and annealed fiber (I) at (a) 100°C with different applied stresses, and with (b) a fixed applied stress (1.6 MPa) but different annealing temperatures. The *s*- and *p*-polarized spectra are offset for better visibility. Note that the data in this figure is the same as the data in Fig. 6 of the article by Perret et al. [1] with the difference that here the *s* (or *p*)-polarized spectra are plotted on top of each other.

Fig. 4a shows the *s*- and *p*-polarized ATR-FTIR spectra of the annealed fiber (I) at fixed temperature, 100°C, and Fig. 4b at fixed annealing stress, 1.6 MPa. Note that all ATR-FTIR data from the Mendeley repository can be plotted with the open source Python package opusFC (https://pypi.org/project/opusFC/).

**Fig. 5:**
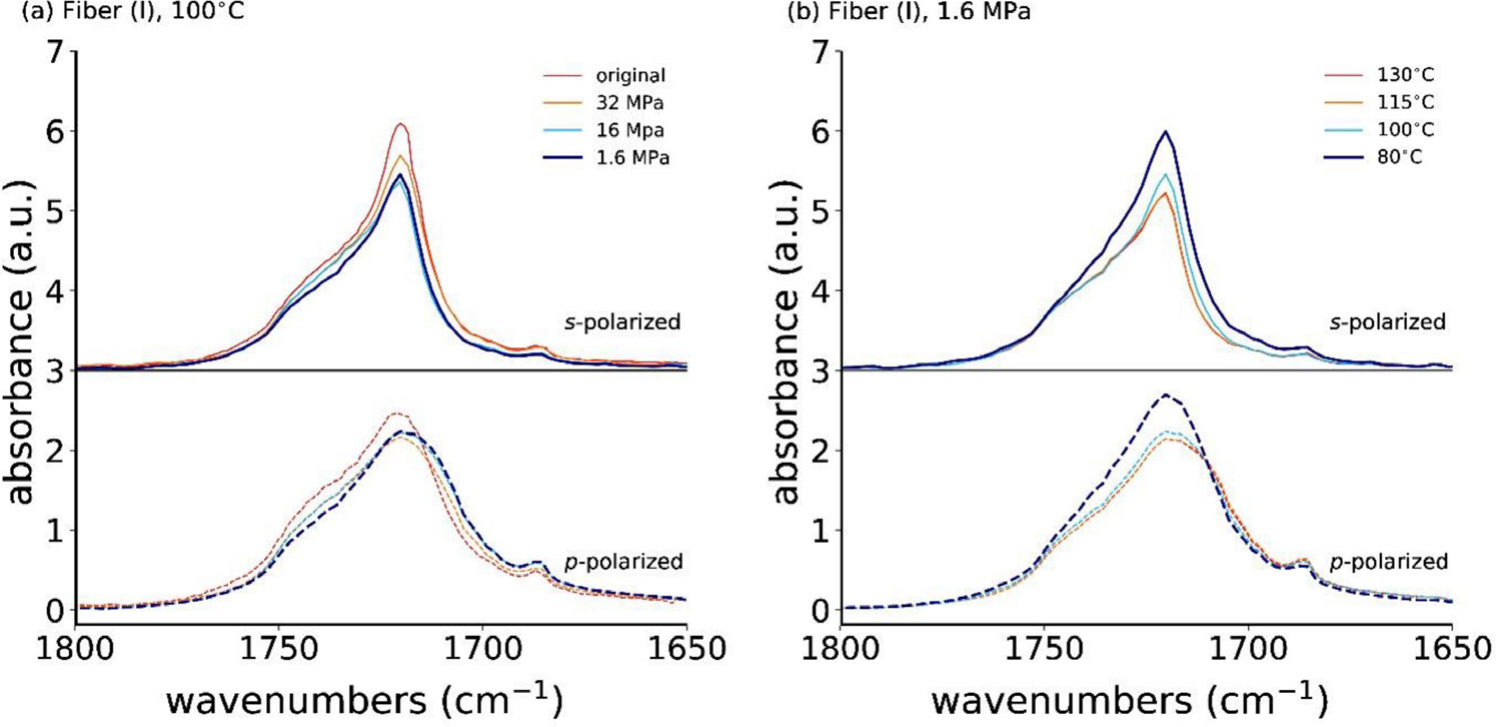
Close-up of polarized ATR-FTIR spectra for the νC=O vibration (α-crystal) for the original and annealed fiber at (a) 100°C with different applied stresses and with (b) a fixed applied stress (1.6 MPa) but varying annealing temperatures.

A close-up of the νC=O vibration (1724 cm^−1^), from the crystalline α-phase, is shown in Fig. 5a for a constant annealing temperature of 100°C, but varying annealing stress, and in Fig. 5b for constant annealing stress of 1.6 MPa, but varying annealing temperature. This band's absorbance is decreasing in the *s*-polarized spectrum with decreasing annealing stress, while the *p*-polarized spectrum is hardly changing (Fig. 5a). Interestingly, the band's absorbance is higher in both, the *s*- and *p*-polarization, for the fibers annealed under low stress at 80°C (Fig. 5b).

**Fig. 6:**
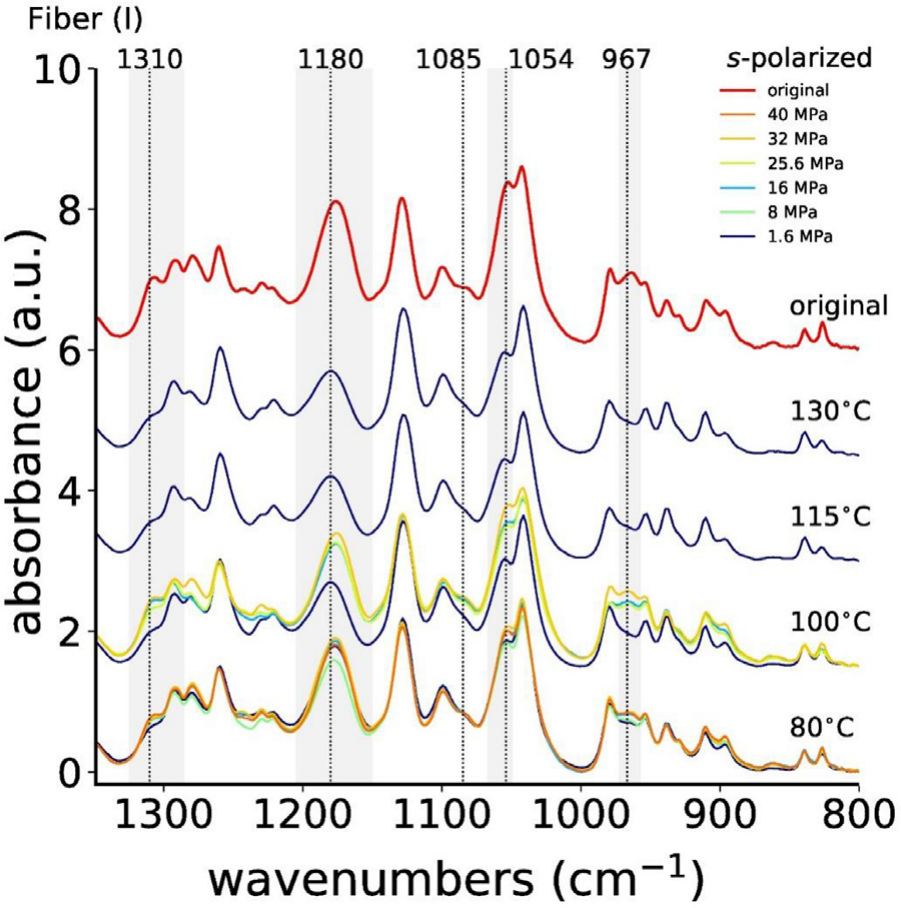
ATR-FTIR spectra, measured with *s*-polarization of all stress-annealed fibers (I).

**Fig. 7:**
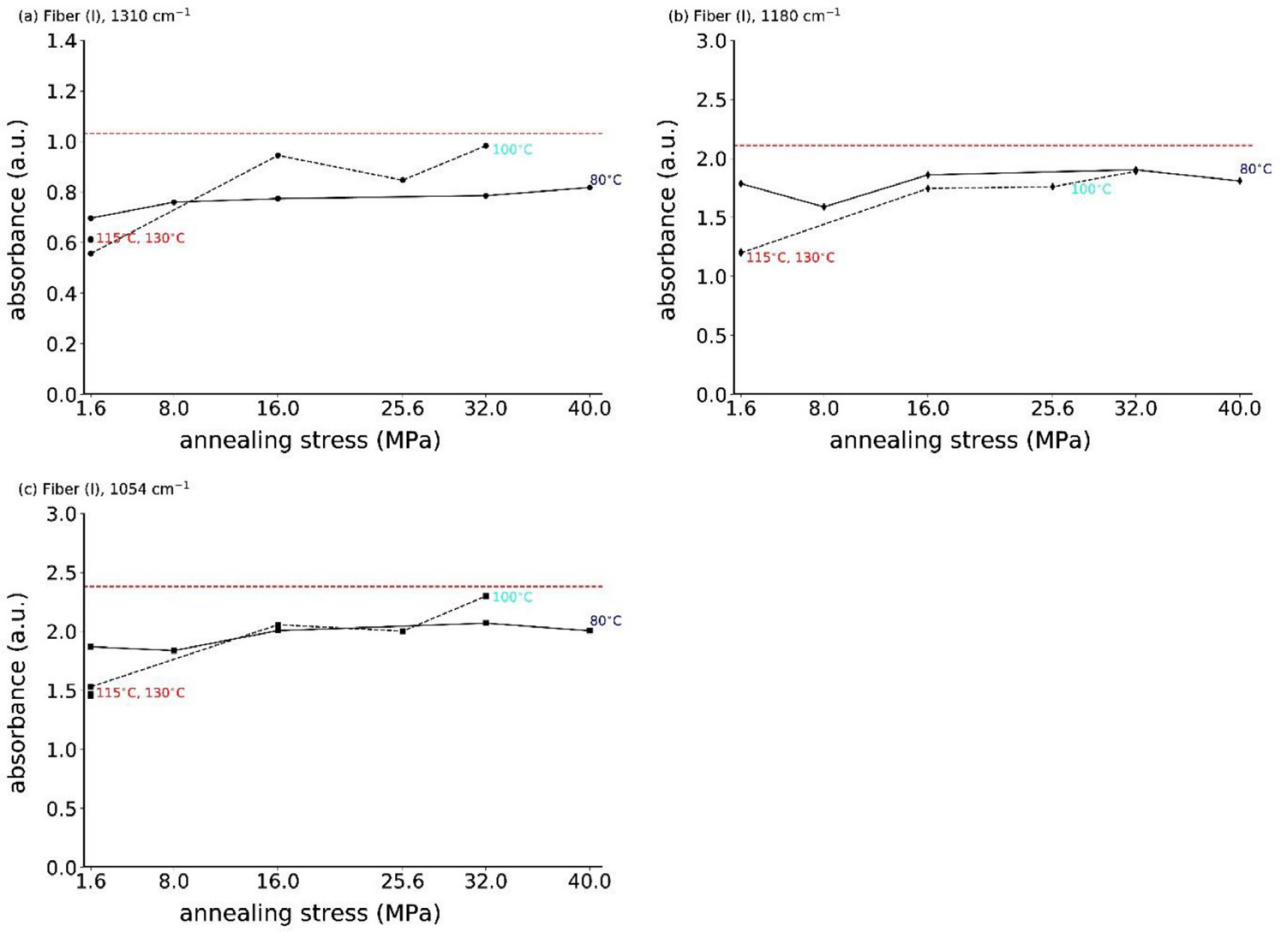
Absorbances of IR bands (a) 1310 cm^−1^, (b) 1180 cm^−1^ and (c) 1054 cm^−1^ as a function of annealing stress for fiber (I). The dashed horizontal red lines are the reference values of the original fiber (I).

Fig. 6 shows all measured *s*-polarized ATR-FTIR spectra of fiber (I), and Fig. 7 shows the absorbances of IR bands 1310, 1180 and 1054 cm^−1^ as a function of the annealing stress.

**Fiber (II)**

**Fig. 8:**
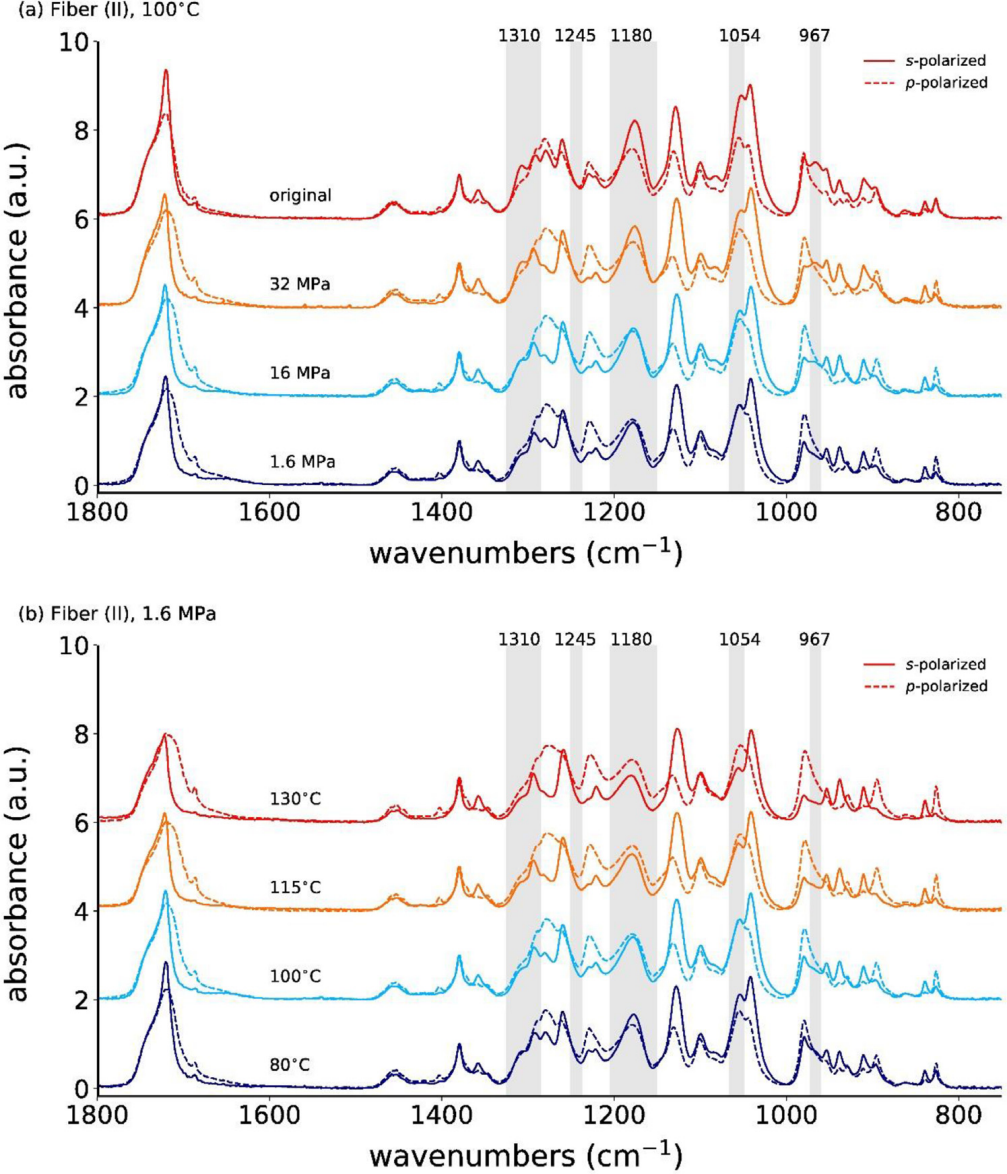
Polarized ATR-FTIR spectra for (a) the as-spun and stress-annealed fiber (II) at 100°C with different applied stresses and (b) for the low-stress (1.6 MPa) annealed fibers (II) at different temperatures. The curves are offset for better visibility.

The *s*- and *p*-polarized spectra for the as-spun and annealed fiber (II) at 100°C, with applied stresses ranging from 1.6 MPa to 32 MPa, are shown in Fig. 8a. Fig. 8b shows the spectra for a constant applied stress of 1.6 MPa, but different annealing temperatures.

**Fig. 9:**
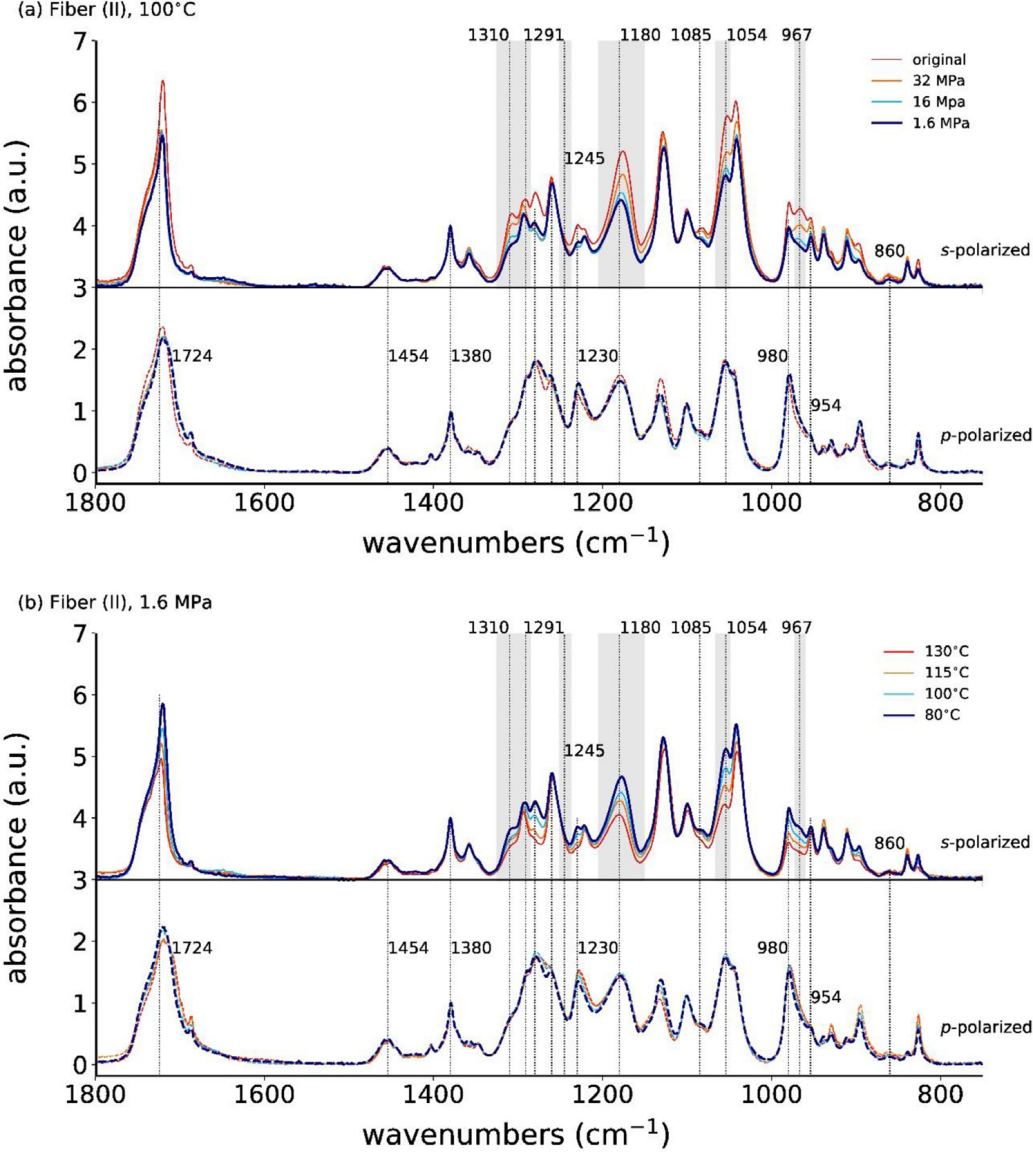
Polarized ATR-FTIR spectra for the as-spun and annealed fiber (II) at (a) 100°C with different applied stresses, and with (b) a fixed applied stress (1.6 MPa) but different annealing temperatures. The *s*- and *p*-polarized spectra are offset for better visibility.

Fig. 9a shows the plots of *s*- and *p*-polarized spectra of the annealed fiber (II) at 100°C for different applied annealing stresses, and Fig. 9b for constant annealing stress (1.6 MPa) but different annealing temperatures. Similar trends as for fiber (I) are observed.

**Fig. 10:**
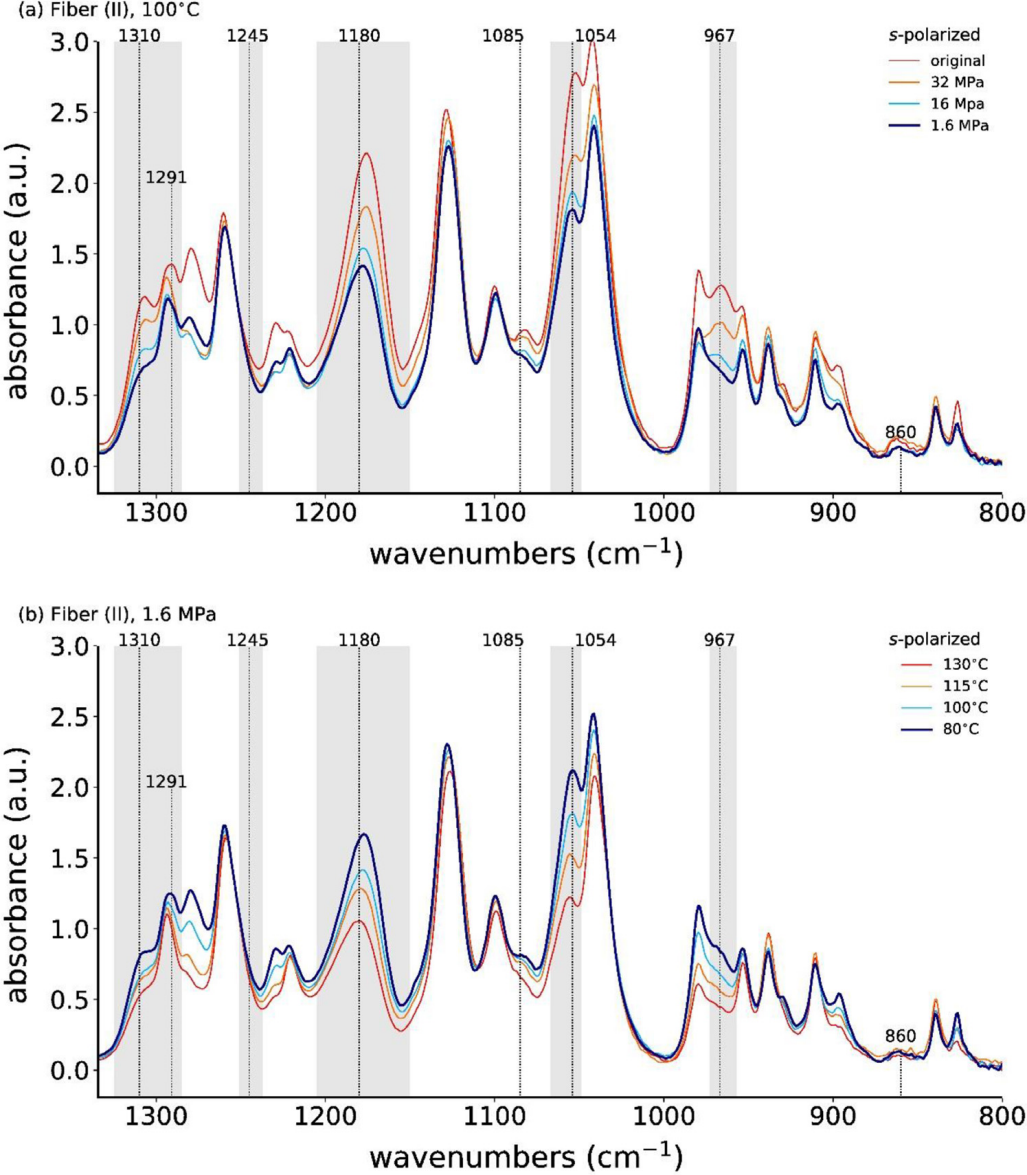
ATR-FTIR spectra with *s*-polarization for the as-spun and stress-annealed fiber (II) at (a) 100°C with different applied stresses, and with (b) a fixed applied stress (1.6 MPa) but varying annealing temperatures.

Close-ups of the s-polarized spectra are shown in Fig. 10.

In the as-spun and high-stress annealed fibers, the absorbances of the *s*-polarized IR bands at 1310, 1180, 1054 and 967 cm^−1^ are higher than in the low-stress annealed fibers.

**Fig. 11:**
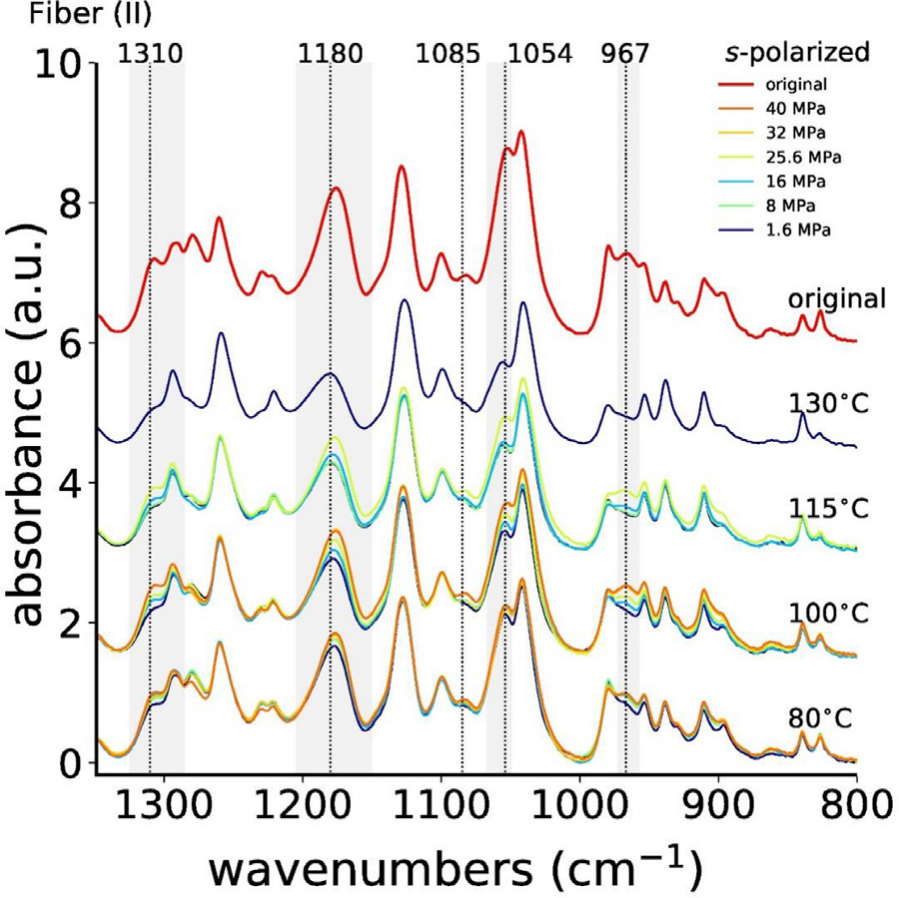
ATR-FTIR spectra, measured with *s*-polarization, of all stress-annealed fibers (II).

**Fig. 12:**
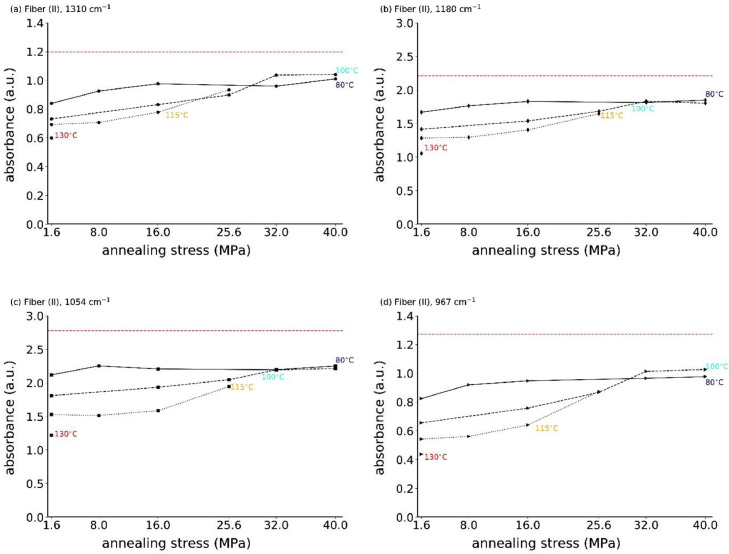
Absorbances of IR bands (a) 1310 cm^−1^, (b) 1180 cm^−1^, (c) 1054 cm^−1^ and (d) 967 cm^−1^ as a function of annealing stress for fiber (II). The dashed horizontal red lines are the reference values of the original fiber (II).

Fig. 11 shows all measured *s*-polarized ATR-FTIR spectra of fiber (I), and Fig. 12 shows the absorbances of IR bands 1310, 1180 and 1054 cm^−1^ as a function of the annealing stress.

#### 1.2.2 In-situ polarized ATR-FTIR under cyclic drawing of P3HB fibers annealed at low stress

**Fiber (I)**

**Fig. 13:**
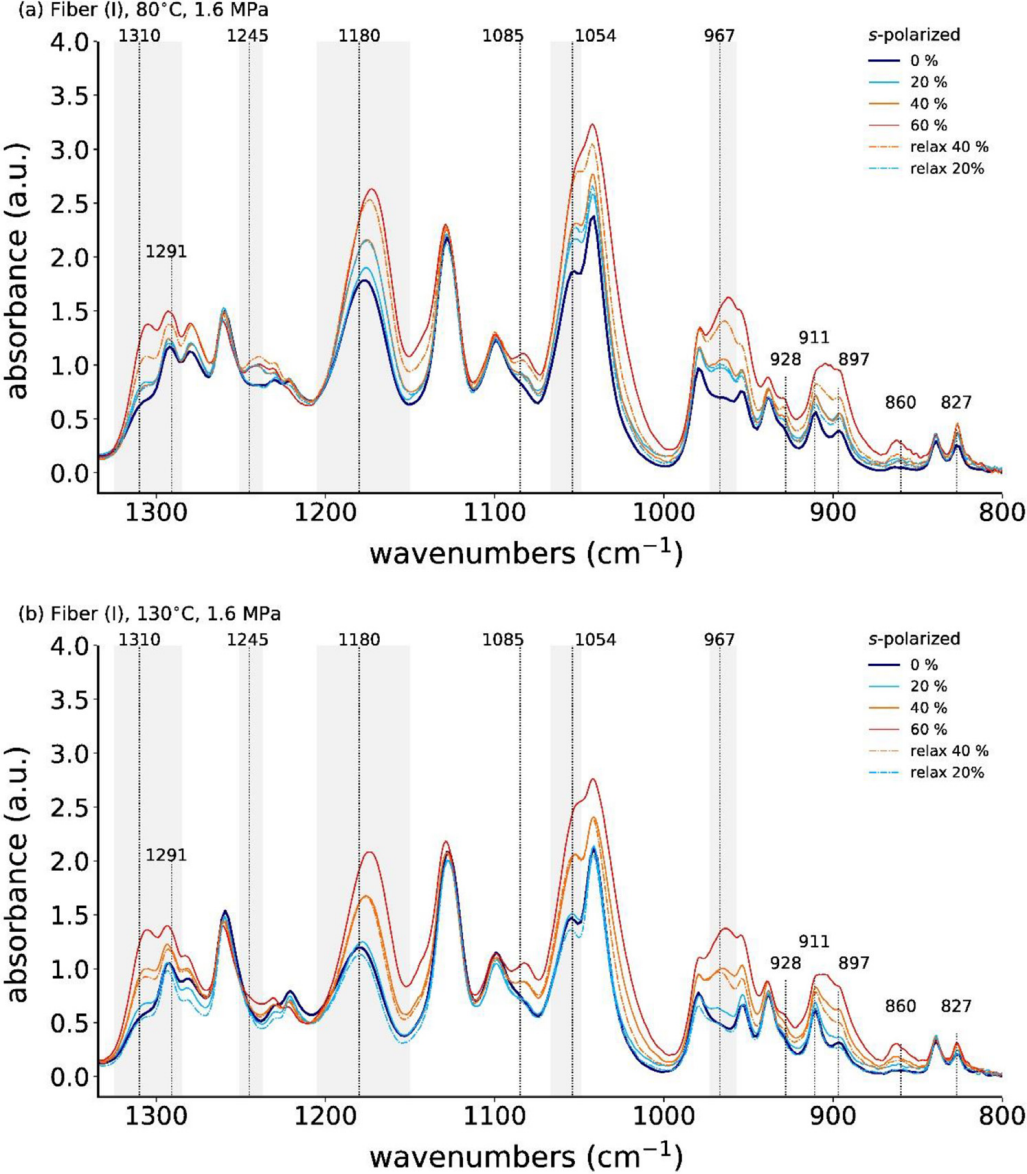
In-situ ATR-FTIR spectra with *s*-polarization during cyclic tensile drawing (one cycle) of P3HB fiber (I), which has been previously annealed at (a) 80°C with 1.6 MPa, and at (b) 130°C with 1.6 MPa.

**Fig. 14:**
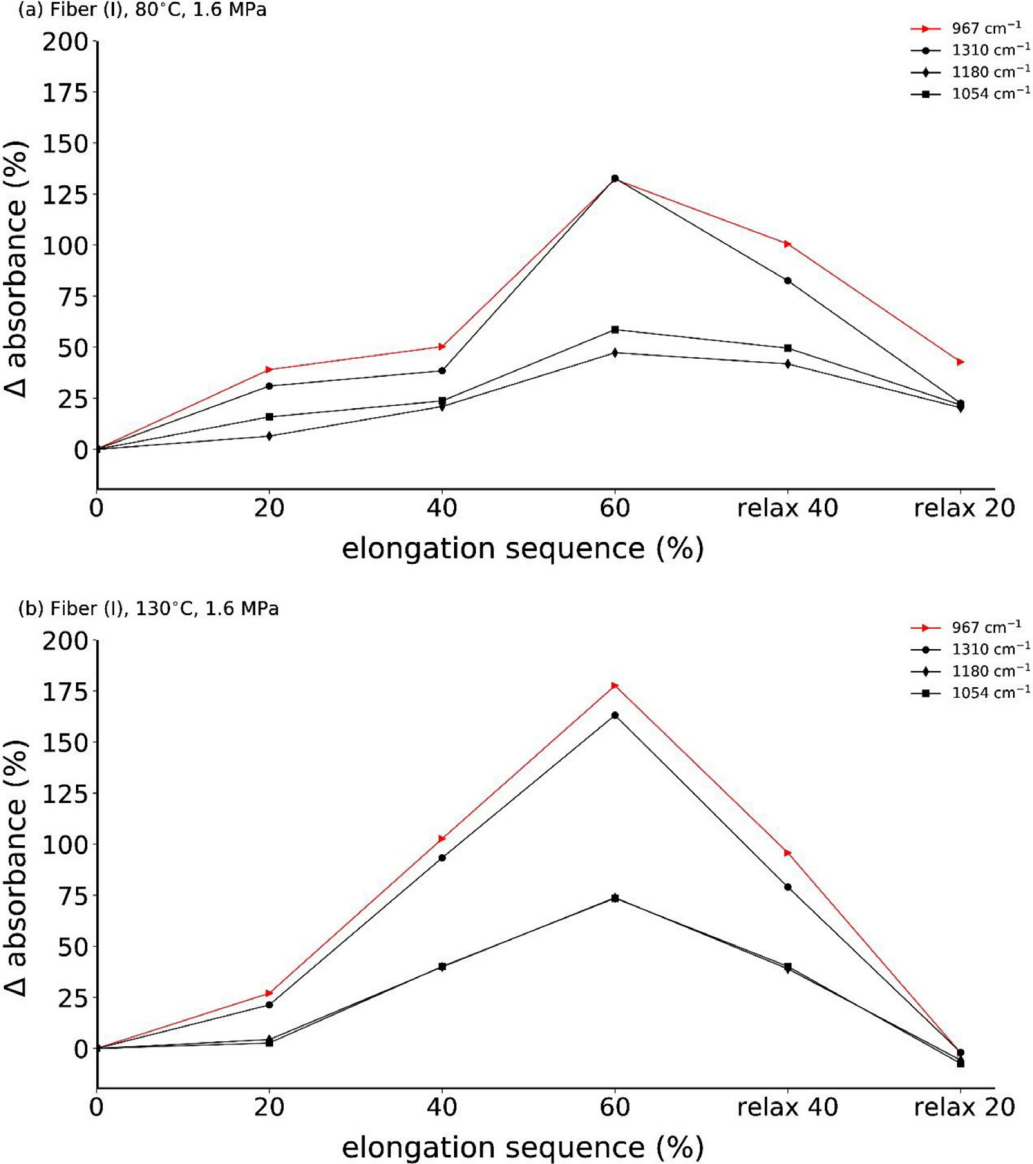
Percentage change in absorbance (with respect to 0% elongation) of mesophase IR bands.

Fig. 13 shows the in-situ *s*-polarized ATR-FTIR spectra of annealed fiber (I) at low stress (1.6 MPa at 80°C and 130°C) during one tensile drawing cycle. Fig. 14 summarizes the corresponding percentage change in absorbance of specific mesophase IR bands.

**Fiber (II)**

**Fig. 15:**
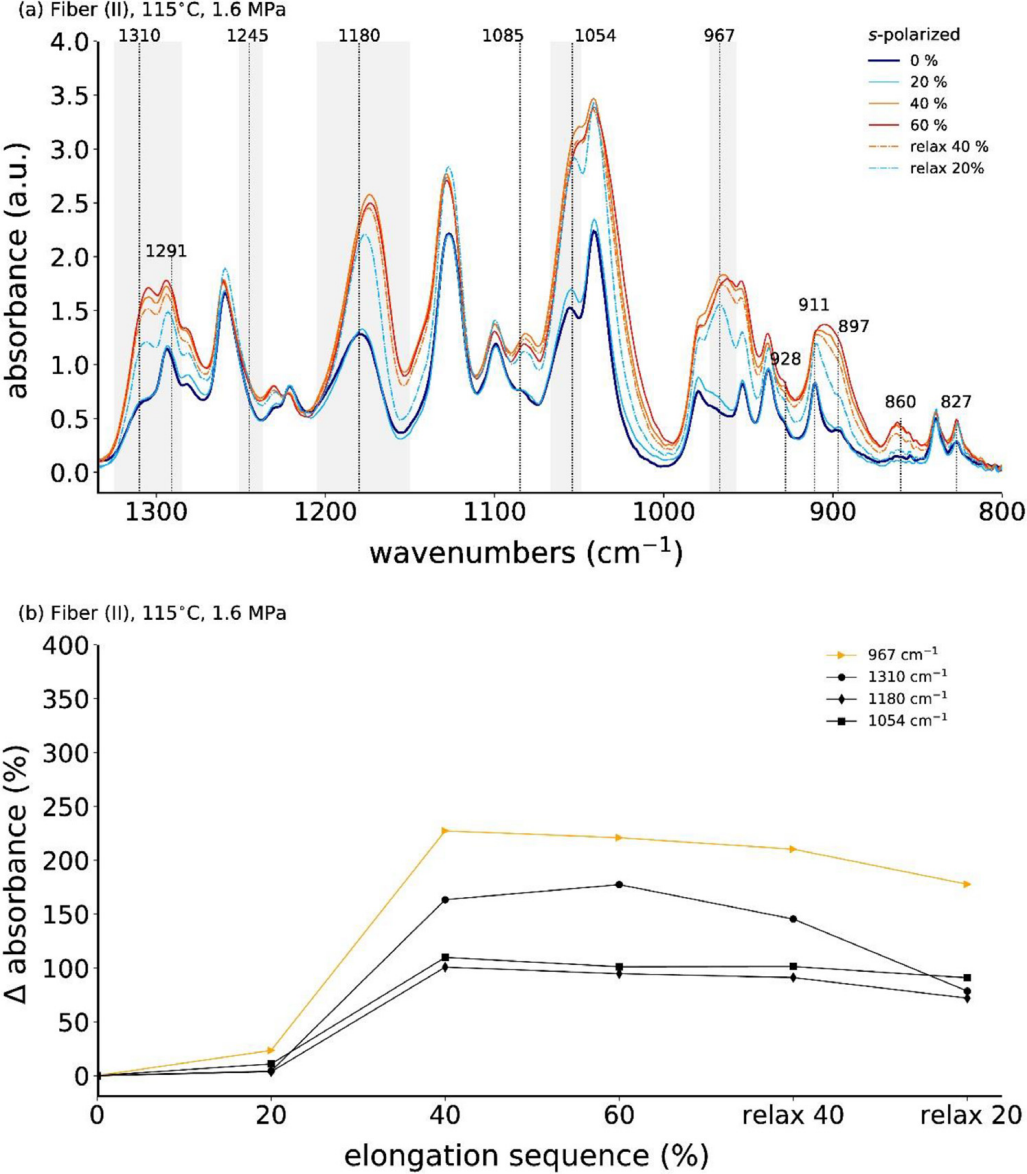
(a) Normalized in-situ ATR-FTIR spectra with *s*-polarization during cyclic tensile drawing (one cycle) of P3HB fiber (II), which has been previously annealed at 115°C with 1.6 MPa, and (b) corresponding percentage change in absorbance (with respect to 0% elongation) of mesophase IR bands as a function of elongation sequence.

Fig. 15 shows the *s*-polarized ATR-FTIR spectra for low-stress annealed (1.6 MPa at 115°C) fiber (II) measured during the first tensile drawing cycle.

### 1.3 DSC analysis

**Fig. 16:**
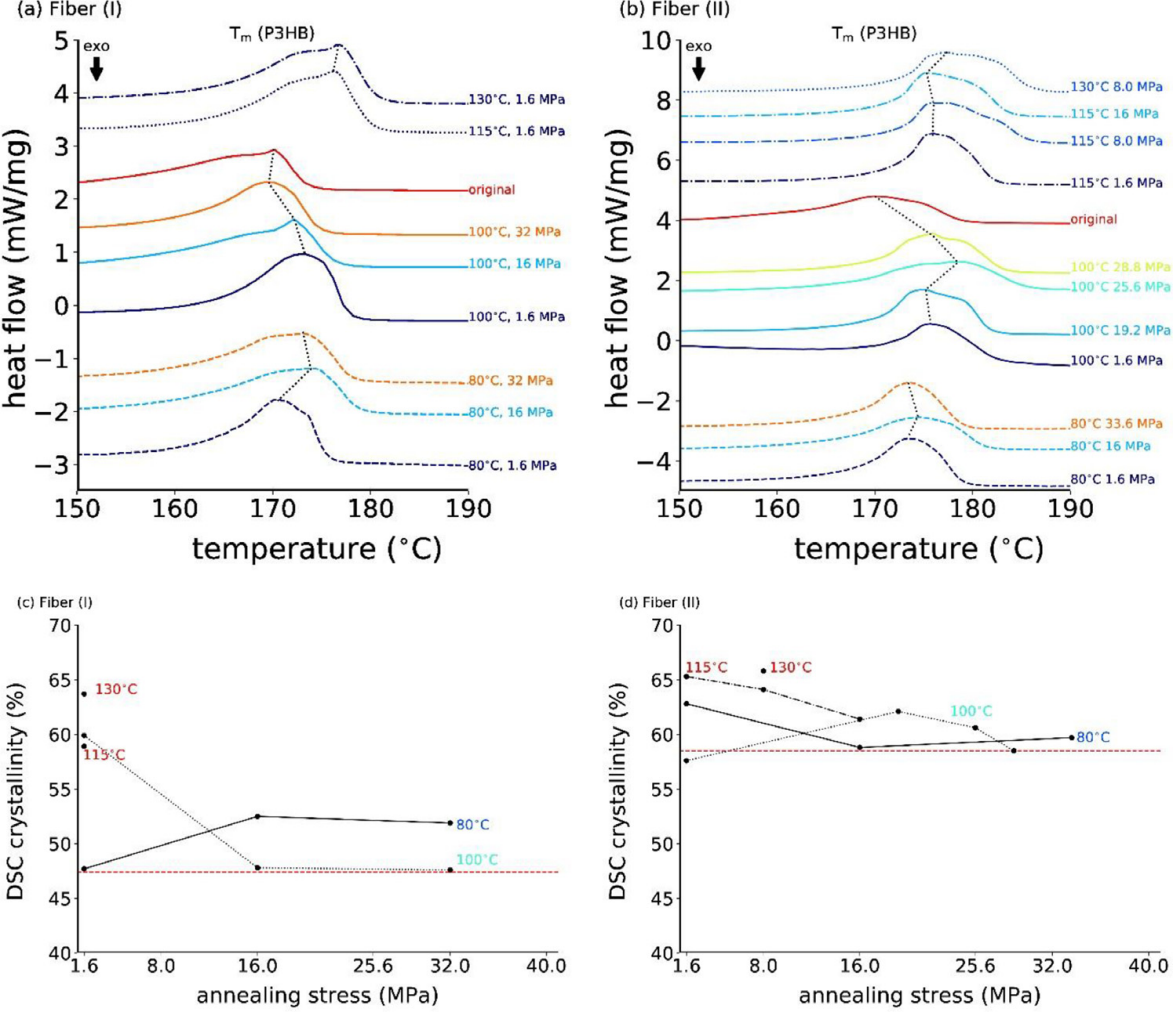
DSC thermograms of original and (a) stress annealed fibers (I), and of (b) stress annealed fibers (II). Melting temperatures corresponding to values in Table 1 are indicated with dashed black lines. Calculated crystallinities from the integrated melting peak are shown in (c) for fibers (I), and in (d) for fibers (II). The dashed horizontal red lines represent the values from the original fibers.

DSC was performed only on a selected number of fibers, since not enough material remained for several annealing conditions after the WAXD and ATR-FTIR studies. Note that all the raw data files are given as (.txt) files in the Mendeley repository. The measured DSC heating curves of P3HB fiber (I) always showed two melting peaks, a small one at about 55°C, which corresponds to the melting of PCL (plasticizer in fiber (I)) [2], [3], [4], [5], and a strong melting peak of P3HB at about 170°C. Fig. 16a,b show the P3HB melting peaks for original and annealed fibers (I) and (II), respectively. Broad shoulders towards lower temperatures are observed for the original fibers (not annealed), and for fibers annealed at high stress, which have a high amount of mesophase [6]. This shoulder is completely absent for the fiber annealed at low stress (no or reduced amount of mesophase), where the melting peak is sharper.

The calculated crystallinities from DSC are plotted vs. the annealing stress in Fig. 16c,d for original and annealed fibers (I) and (II), respectively. The melting temperatures (positions of peak maxima) of P3HB, the melting enthalpies and the calculated crystallinity values are summarized in Table 1. The melting temperatures, as well as the crystallinities, mostly increase with decreasing annealing stress and increasing annealing temperature. The original fiber (II) has a higher crystallinity than fiber (I), since the used compound for fiber (II) was more pure. Most-likely the additives in fiber (I) hinder the crystallization of P3HB.

**Table 1:** Thermal properties and calculated crystallinity values of original and stress-annealed fiber samples.

Samples	*T*_m_ (P3HB) (°C)	$\Delta H_{m}$(J/g)	χ (%)
Fiber (I), original	170.1	68.7	47.4

Fiber (I), 130°C, 1.6 MPa	176.7	92.4	63.7
Fiber (I), 115°C, 1.6 MPa	176.2	85.4	58.9

Fiber (I), 100°C, 32 MPa	169.6	62.5	43.1
Fiber (I), 100°C, 16 MPa	172.2	64.4	44.4
Fiber (I), 100°C, 1.6 MPa	173.3	72.5	50.0

Fiber (I), 80°C, 32 MPa	173.1	75.2	51.9
Fiber (I), 80°C, 16 MPa	173.9	76.1	52.5
Fiber (I), 80°C, 1.6 MPa	170.5	69.2	47.7

Fiber (II), original	170.0	84.9	58.5

Fiber (II), 130°C, 8.0 MPa	177.4	95.3	65.8
Fiber (II), 115°C, 16 MPa	178.3	89.1	61.4
Fiber (II), 115°C, 8.0 MPa	176.0	93.0	64.1
Fiber (II), 115°C, 1.6 MPa	175.9	94.7	65.3

Fiber (II), 100°C, 28.8 MPa	175.9	84.8	58.5
Fiber (II), 100°C, 25.6 MPa	178.5	87.9	60.6
Fiber (II), 100°C, 19.2 MPa	175.2	90.1	62.1
Fiber (II), 100°C, 1.6 MPa	175.7	83.4	57.6

Fiber (II), 80°C, 33.6 MPa	173.4	86.6	59.7
Fiber (II), 80°C, 16 MPa	174.4	85.2	58.8
Fiber (II), 80°C, 1.6 MPa	173.4	91.0	62.8

## 2 Experimental Design, Materials and Methods

The materials and the description of experimental methods to measure the structure of stress-annealed P3HB fibers have already been partially described in the article by Perret et al. [1]. Part of the text below has been taken from the said article and is therefore put into quotes.

### 2.1 Materials

"Two P3HB fibers were melt-spun from modified P3HB (density 1.2 g/cm3), provided by Biomer (Krailling, Germany), on a customized pilot melt-spinning plant originally built by Fourné Polymertechnik (Alfter-Impekoven, Germany). Fiber no. 974 (fiber label given by Empa, St. Gallen, Switzerland) was melt-spun from a pelletized P3HB compound (Mw=0.5 MDa), whereas raw P3HB powder (Mw=1.6 MDa), mixed with 20 wt.% plasticizer (tri-nbutyl citrate (TBC)), was used to melt-spin fiber no. 1108. The P3HB pellets, used for fiber 974, contained a nucleating agent (boron nitride (BN)), 20 wt% TBC and various other processing aids, including low molecular weight poly-ε-caprolactone (PCL). The fiber diameters are about 90 μm for both fibers. Fiber 974 was melt-spun with a draw ratio (DR) of 7.0, and fiber 1108 with DR = 6.0. More detailed information about materials, properties and melt-spinning parameters can be found in our previous publications [3], [4], [5]. We label fiber 974 with (I) and fiber 1108 with (II) in the remainder of this article."

### 2.2 Stress-annealing

“The stress-annealing of aged (eight years at 23°C) P3HB fibers was carried out by attaching different weights (1g, 5g, 10g, 16g, 20g, 25g) to the fibers, corresponding to applied stresses of 1.6, 8.0, 16.0, 25.6, 32.0 and 40.0 MPa. The annealing was performed in a furnace with hot air circulation at different temperatures (80, 100, 115, 130°C) for 60 minutes. Above 100 and 115°C, respectively, fibers (I) and (II) were only annealed at low stress (1.6 MPa). Applying higher stresses at elevated temperatures resulted in fiber breakage within a few minutes. The length of each monofilament was measured at room temperature, with an applied weight of 1g (pre-stress of 1.6 MPa), before and after annealing, in order to determine the change in filament diameter and thus the linear mass density.” For further details we refer the reader to the article by Perret et al. [1].

### 2.3 Synchrotron WAXD

“In-situ WAXD measurements were performed at the cSAXS beamline at the Swiss Light Source synchrotron of the Paul Scherrer Institute in Switzerland. Differently annealed monofilaments have been mounted on a sample holder (horizontally) and WAXD patterns were measured at the center of the fibers with 5 second exposures using a Pilatus 2M detector [7]. The sample to detector distance was 31.9 cm. The x-ray beam was focused with mirrors to a spot size of about 10 µm (perpendicular to fiber axis) and its energy was set to 11.792 keV.” For further details we refer the reader to the article by Perret et al. [1].

### 2.4 Polarized ATR-FTIR

“Polarized ATR-FTIR spectra have been recorded from differently stress-annealed P3HB fibers with a Bruker Tensor 27 FTIR spectrometer (Bruker Optics, Ettlingen, Germany), using a single reflection attenuated total reflectance (GladiATR™) accessory from Pike Technologies (Fitchburg, Wisconsin, United States). The FTIR spectrometer uses a mid-infrared (MIR) Globar source and a narrow-band mercury cadmium telluride (MCT) detector. The ATR accessory is equipped with a monolithic diamond ATR crystal but does not have the option to add polarizers.” The setup modifications and fiber holders are described in the following sections. For further details we refer the reader to the article by Perret et al. [1].

#### 2.4.1 GladiATR^TM^ setup modifications for polarized measurements

**Fig. 17:**
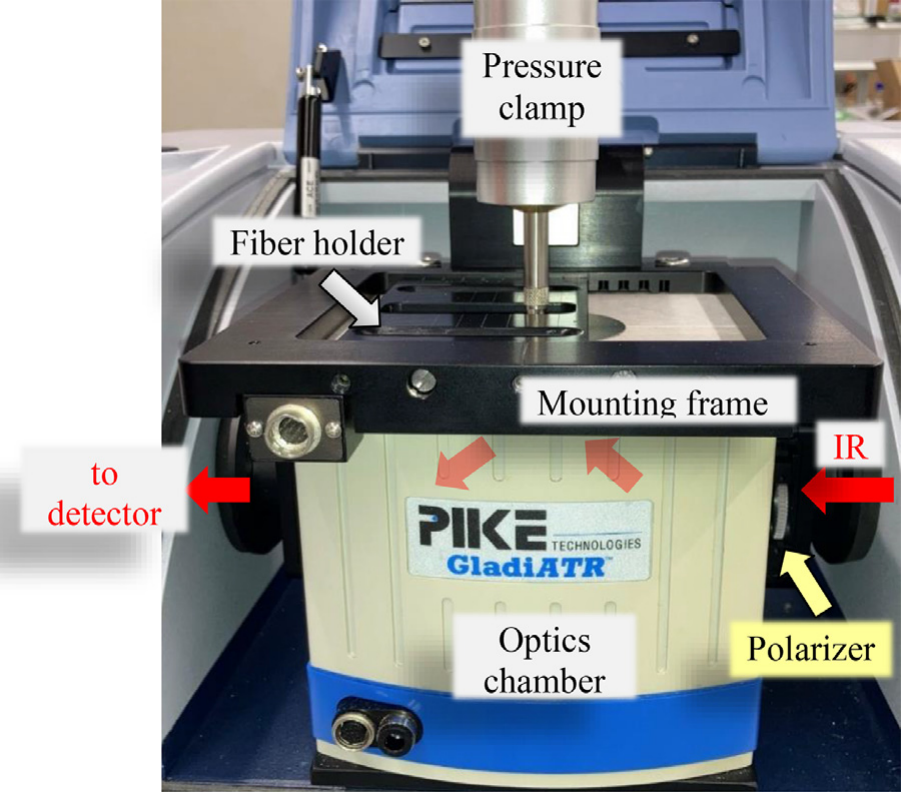
GladiATR^TM^ from Pike Technologies (Fitchburg, Wisconsin, United States) with custom-built polarizer mount, fiber holder and mounting frame.

The GladiATR^TM^ from Pike Technologies (Fitchburg, Wisconsin, United States) was modified in order to be able to make polarized measurements. The added polarizer slot is shown in Fig. 17.

#### 2.4.2 ATR-FTIR fiber holder for static measurements

**Fig. 18:**
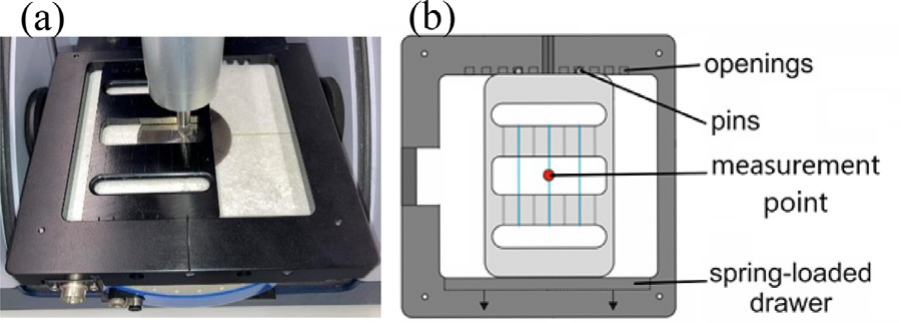
(a) Picture and (b) schematic of ATR-FTIR fiber holder for static measurements.

Fig. 18 shows the fiber holder for static polarized ATR-FTIR measurements.

#### 2.4.3 ATR-FTIR fiber holder for cyclic tensile drawing

**Fig. 19:**
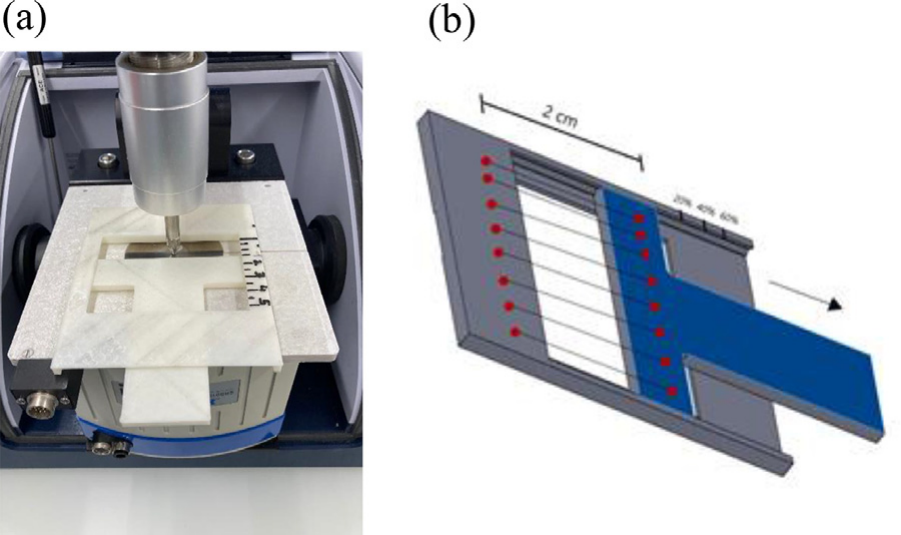
(a) Picture and (b) schematic of ATR-FTIR holder for measurements during cyclic tensile drawing.

Fig. 19 shows the fiber holder for polarized ATR-FTIR measurements during cyclic tensile drawing.

### 2.5 DSC

The thermal properties and average crystallinity values of the P3HB fibers were determined using DSC. All measurements were carried out in nitrogen atmosphere (40 mL/min) using the instrument DSC 214 Polyma (Netzsch, Selb, Germany). The fibers were chopped into small pieces (∼2 mm long), and about 5-10 mg of cut fibers were put into small crucibles. The crucibles were then heated from 25°C to 200°C and cooled from 200°C to 25°C at a ramping rate of 10°C/min. The data analysis was carried out using the NETZSCH Proteus thermal Analysis software (Version 7.1.0, Netzsch, Selb, Germany).

The melting peaks of P3HB have been integrated in order to calculate the P3HB crystallinities from the melting enthalpies, $\Delta H_{m}$, according to Eq. 1. The heat enthalpy, $\Delta H_{\text{ref}}$, of an ideal P3HB crystal was taken to be 145 J/g [8].

(1) $$\chi \left(\%\right)=\frac{\Delta H_{m}}{\Delta H_{\text{ref}}}\;\cdot \;100$$

## Ethics Statement

The authors followed generally expected standards of ethical behavior in scientific publishing throughout article construction.

## CRediT Author Statement

**E. Perret:** Software, Formal analysis, Investigation, Data curation, Writing original draft,Visualization. **K. Sharma:** Data curation. **S. Tritsch:** Data curation. **R. Hufenus:** Supervision,Project administration, Writing review & editing.

## Declaration of Competing Interest

The authors declare that they have no known competing financial interests or personal relationships which have, or could be perceived to have, influenced the work reported in this article.

## References

[1] Perret E. (2021). Reversible mesophase in stress-annealed poly(3-hydroxybutyrate) fibers: a synchrotron x-ray and polarized ATR-FTIR study. Polymer.

[2] Selli F. (2020). Mesophase in melt-spun poly(ɛ-caprolactone) filaments: structure–mechanical property relationship. Polymer.

[3] Perret E. (2020). Structural response of melt-spun poly(3-hydroxybutyrate) fibers to stress and temperature. Polymer.

[4] Perret E. (2019). Tensile study of melt-spun poly(3-hydroxybutyrate) P3HB fibers: Reversible transformation of a highly oriented phase. Polymer.

[5] Hufenus R. (2015). Molecular orientation in melt-spun poly(3-hydroxybutyrate) fibers: Effect of additives, drawing and stress-annealing. Eur. Polym. J..

[6] Perret E., Hufenus R. (2021). Insights into strain-induced solid mesophases in melt-spun polymer fibers. Polymer.

[7] Henrich B. (2009). PILATUS: a single photon counting pixel detector for X-ray applications. Nucl. Instrum. Methods Phys. Res. Sect. A.

[8] Czerniecka-Kubicka A. (2017). Thermal properties of poly(3-hydroxybutyrate) modified by nanoclay. J. Therm. Anal. Calorim..

